# Choosing and Managing Aged Care Services from Afar: What Matters to Australian Long-Distance Care Givers

**DOI:** 10.3390/ijerph182413000

**Published:** 2021-12-09

**Authors:** Kate M. Gunn, Julie Luker, Rama Ramanathan, Xiomara Skrabal Ross, Amanda Hutchinson, Elisabeth Huynh, Ian Olver

**Affiliations:** 1 Department of Rural Health, University of South Australia, Adelaide, SA 5000, Australia; xioyen@gmail.com; 2 Allied Health and Human Performance, University of South Australia, Adelaide, SA 5000, Australia; Julie.Luker@unisa.edu.au; 3 Commission on Excellence and Innovation in Health, Adelaide, SA 5000, Australia; Rama.Ramanathan@sa.gov.au; 4 Justice and Society, University of South Australia, Magill, SA 5072, Australia; Amanda.Hutchinson@unisa.edu.au; 5 Department of Health Services Research & Policy, The Australian National University, Acton, ACT 2601, Australia; elisabeth.huynh@anu.edu.au; 6 School of Psychology, The University of Adelaide, Adelaide, SA 5000, Australia; ian.olver@adelaide.edu.au

**Keywords:** aging, aged, decision making, health care quality, communication, housing for the elderly, health services for the aged

## Abstract

This research aims to identify the factors that influence caregivers’ decisions about the aged care providers they select for their elder relatives when caring from a distance and what they value once they have engaged a service. Adult long-distance carers for older relatives living within Australia were purposively sampled and they participated in audio-recorded interviews. A thematic analysis was employed to investigate the data. A sample of 13 participants enabled data saturation with no new major themes identified in the final three interviews. Participants were 50 to 65 years (Mean = 59.8) and mostly (77%) female. Four themes emerged relating to selection of care providers: (1) availability of care, (2) financial arrangements, (3) proximity and location, and (4) reputation of care provider. Five themes detailed valued qualities of care: (1) vigilant monitoring and responsivity, (2) communication with family, (3) flexibility and proactiveness of care, (4) staffing, and (5) access to appropriate and holistic care to maintain wellbeing. Long-distance caregivers face barriers in selecting and managing aged care services from afar within a complex Australian aged care system. They strongly value regular, proactive communication about the wellbeing of their relatives and may be particular beneficiaries of communication and assistive monitoring technologies.

## 1. Introduction

Aged care involves the provision of care for older people in their own homes or institutions (aged care facilities). The Australian aged care system has been described as being complex and difficult to navigate [1]. This is likely to be exacerbated when carers are trying to select and manage aged care services for their relatives remotely. To the best of our knowledge, the number of Australians caring from a distance is currently unknown. In 2006, in the United States, 5–7 million (15%) people were long-distance caregivers of elderly relatives. This number was projected to double by 2020. Due to COVID-19 restrictions, it is likely that intra-/inter-state and overseas relocations dramatically reduced during 2020 and 2021, and this impact may persist for some time. However, these restrictions have also meant that many carers who would normally make visits in person to arrange care, have been forced to make those decisions remotely.

Understanding the experiences and perspectives of aged care users and their lon-distance carers is especially relevant in the context of the recent introduction of a consumer-driven aged care model in Australia [2], in-line with the 2019 Australian Federal Government’s Aged Care Quality Standards [3], which has given consumers more choice about the care they receive. In Australia, in 2018, 35% of people aged ≥65 years (3,900,000) lived in their own homes and accessed Australian Government subsidized aged care services to assist with their everyday activities [4], such as home cleaning; access to medical care; and help with showering, dressing, and toileting [5]. Access to government-subsidized aged care services (in private homes or residential care facilities) is even higher near the end of life. Eighty percent of Australians (≥65 y/o) who passed away between 2010 and 2011 accessed government-subsidized aged care services (at home or in residential care) in the previous eight years [6].

Recent efforts have been made to improve the assessment of the quality of aged care in Australian institutions [7]. Autonomy, care, respect, and person-centeredness are key aspects that previous research has shown tends to guide Australian older people and their carers’ selection of aged care providers [8]. International research has identified additional aspects of aged-care institutions (e.g., characteristics of the care staff and environment, physical, and social aspects of care) that are important to older residents [9,10]. However, few studies have focused specifically on long-distance caring.

Available research on long-distance care for older people provides insights about the challenges experienced by carers, but there is a lack of evidence, particularly from the Australian context, on what these remote carers consider important when making or contributing to decisions about aged care providers for their elder relatives [11]. The greater the distance that carers live from their elder relatives, the greater the likelihood of challenges related to long distance caregiving. These may be financial, emotional, communication-related, and/or psychological in nature [11,12]. For example, to perform this role, carers may have to miss out on work, not visit their elder relatives as frequently as desired, and/or spend many hours organizing and monitoring care from a distance [13]. Long distance carers are also known to be less likely to maintain strong bonds with the people they are caring for and may experience guilt, regret, and frustration at being less able to participate in shared decision-making between service providers and their relatives [14].

Families have consistently expressed the need to be more involved in the care of their elder relatives and to have more frequent communication with their professional carers [15]. Communication-related challenges are thought to be exacerbated when distance divides the family and their elder relatives, which can lead to significant levels of stress in carers [11]. Challenges with communication may also result in decisions being made about care that do not align with the desires of both parties.

This research aims to identify the factors that influence Australian long-distance caregivers’ decisions about the aged care providers they select for their elder relatives (who live at a distance but within Australia), and what they value once they have engaged a service. This work will assist families and caregivers to discriminate between the different competing care options and will reveal the difficult trade-offs that need to be made when navigating what is often described as a complex, variable, and fragmented Australian aged care system [16]. It may also assist aged care providers to align their provision of services with consumers’ perspectives, to improve their competitiveness, better orientate services towards the needs of remote carers, and ensure the best outcomes for those receiving care. Given COVID-19 social distancing and travel restrictions, these lessons are particularly timely due to the increased number of people facing the challenges of caring for an elderly relative remotely. 

## 2. Materials and Methods

As detailed below, this study was underpinned by a ground up, descriptive qualitative framework [17,18], and thematic analysis methods were employed to analyze the data [19,20,21]. Ethical approval was obtained from the Human Research Ethics Committee, University of South Australia (#0000036294). 

### 2.1. Study Sample

Participants were adults who (1) self-identified as being the key decision maker about care for an older relative receiving aged care services; (2) lived at a long distance from that relative (100 km or more), within Australia; (3) were unable to travel to be involved in key decisions about their family member’s care from an aged care provider in person (due to social, economic, or geographic constraints) at least some of the time; (4) were not severely cognitively impaired; and (5) spoke English.

### 2.2. Participant Recruitment

We sampled a diverse range of participants including family carers living rurally, intra-, and inter-state; participants with relatives receiving community aged care services in their own home; and some with experience of relatives living in residential aged care facilities. Recruitment occurred through purposive sampling initially. Key personnel at a large private provider of aged care services in South Australia identified potential participants who met the study’s selection criteria, and provided them with written information about the study, consent forms, and an invitation to contact the researchers if they wished to participate. Recruitment was slower than anticipated so snowball sampling and promotion of the study in newsletters were additional strategies employed. Participation was completely voluntary (no reimbursement provided). 

The researchers sent written information about the study to 24 people who expressed interest in participating. Of these, seven did not return consent forms for participation, and four were willing to participate but did not meet all of our inclusion criteria. We anticipated interviewing 15–20 participants, but ceased recruitment once data saturation was reached. We defined data saturation as the stage of analysis where no new main themes had been obtained from the last three interviews analyzed. Saturation point was achieved with a sample size of 13 participants, indicating that adequate data had been collected to answer our research questions and no new insights were being gained.

### 2.3. Enquiry Framework, Data Collection, and Management

We used a qualitative, ground up, descriptive framework approach for this study. Key questions addressed were as follows:

1. What factors were considered/choices made when deciding on an aged care package and aged care provider? (Planning phase)
2. What you would like more or less of from the aged care package and aged care provider, now that care is being delivered? (Maintenance phase)

Additional prompts were used to encourage discussion about issues particularly relevant to caring from a distance (and therefore not as physically present during care planning processes). The interview guide, developed by the authors following consultation with the literature and consumers, is provided in the Appendix A. Interviews were conducted by telephone by JL (female, experienced qualitative researcher, and physiotherapy) and KG (female, experienced qualitative researcher, and clinical psychologist) between December 2017 and October 2018, at a time that was convenient to participants. It should be noted that this was prior to the COVID-19 pandemic. One researcher led the interview and a second researcher listened and took interview notes. Where an interviewer had a previous relationship with a participant, the other interviewer took the lead and the conflicted interviewer offered to sit out of the interview (although no participants agreed to this). Neither interviewer had competing interests in the research topic. Interviews were audio recorded, and later transcribed verbatim by a professional service. Interview notes were summarized according to the two key research questions outlined above. Basic demographic and contextual data were collected during the interview and were added to an Excel spreadsheet so that the study’s sample could be described. Two participants emailed additional, unsolicited information on thoughts they had not mentioned in the interview but felt were important. These were analyzed together with the relevant transcripts.

### 2.4. Analysis

Following a thematic analysis approach [19,20,21], data were organized with assistance from NVivo software (NVivo 12, QRS International Pty Ltd., Burlington, MA, USA) to aid in the organization of the data. This happened in five stages: familiarization with the data (listening to interviews, reading transcripts and notes); coding the data; clustering the codes into coherent, meaningful groups in a hierarchical tree structure; and finally forming descriptive themes and subthemes. We employed inductive data coding and descriptive thematic development. We interpreted the data with minimal inference, in order to present a rich description of participants’ experiences or perspectives in everyday language [20,21], based on the essentialist assumption that the descriptions participants provided were direct insights into their experiences.

To ensure rigor, discussions and consensus decisions by two researchers (JL and KG) occurred at all of the analytical stages. The process of identifying important concepts gathered from participants commenced at the conclusion of each interview with a discussion between the two researchers. Codes were allocated to small segments of meaning in the transcripts notes, resulting in 151 initial codes. Using an iterative process of constant comparison between transcripts, the codes were then clustered into coherent, meaningful groups in a hierarchical tree structure, forming 9 descriptive themes and 23 subthemes. We also aligned interview quotations to each theme to ensure we stayed close to the original data. Additional examples from the dataset that support the thematic findings can be found in the Supplementary Table (Table S1). Reporting was informed by the Consolidated Criteria for Reporting Qualitative Research (COREQ) [22]. 

Data from participants and aged care providers were de-identified prior to reporting. A level of data saturation was reached when no new main themes emerged from the final three interviews analyzed. 

## 3. Results

The characteristics of the sample and their elder relatives are provided in Table 1. All participants described aged care arrangements that related to one or both parents living within Australia (therefore the terms ‘parents’ and ‘relatives’ are used interchangeably herein). Distances between carer participants and elderly relatives ranged from 384 to 3214 km; the average distance between them being 1364 km.

### 3.1. Themes

Our analysis identified nine themes, each with sub-themes that related to our research questions (see the thematic tree in Table 2). Four themes aligned principally to the framework category of “deciding on a provider” and five themes aligned to the “qualities of care” families sought from providers during the maintenance phase of their engagement with aged care service providers. Interrelating themes were noted with some data relevant to more than one theme, thus illustrating a complex picture of participants’ experiences of caring for their parents from a distance. Additional interview data that support the themes are available in the Supplementary Table (Table S1).

### 3.2. Factors That Influence Decisions on an Aged Care Service Provider

#### 3.2.1. Availability of Care

The need for residential aged care was frequently precipitated by the rapid deterioration and/or acute hospitalization of an elderly parent and the subsequent time pressures being placed on the family and services to find a nursing home placement or home care provider, so as to enable discharge from the acute setting. Finding available aged care facilities was described by participants as being stressful due to the limited choices, especially in rural areas. Visiting the facilities and meeting care providers in person was considered valuable where possible in order to avoid poor choices and to prevent the need to re-locate their elderly relatives to a more suitable place in the future. The long waiting lists for community care packages (i.e., packages that offer planned and coordinated health and support services to older Australians in their own homes) also meant delays in having access to the care that was required; instead, families were forced to take what they could get, which they often felt was inadequate.

“I just could not find spaces anywhere and she [hospital discharge officer] found a place, they are pretty tough on you, she told us that the first place that came up we would just have to take it and we resisted a fair bit on that, but we said it really mattered especially what location mum was in and she was tough on us and said ‘I am sorry but’… I guess we did not really have much choice, we were lucky in what was offered to us”.

(C-14)

#### 3.2.2. Financial Arrangements

The financial position of the participants and their older parents, along with the financial arrangements offered by the care providers, played an important role in the selection of nursing homes and care plans. 

##### Cost

Many families could not afford their preferred option due to the high costs involved.

“I think a lot of it was based on cost as well, because my mother is not particularly well-off financially, and so cost would have had a factor there too. So, I think that might have been the deciding factor”.

(C-17)

##### Suitable Payment Options

Challenges related to making payments to aged care providers were highlighted by participants. For example, issues with some billing processes caused anxiety in older parents and required the involvement of family members. Additionally, the large sum of money needed for admission to aged care facilities was also a concern for participants. Many sought professional assistance to manage financial arrangements.

“We had to find a large sum of money, which will be refundable when Dad dies… So my sister had to communicate with their solicitor and their financial adviser to sort of shuffle their finances around”.

(C-23)

##### Value for Money

Participants wanted to ensure that service providers were able to meet their older parents’ needs at an affordable cost. This applied to both nursing homes and community care packages.

“I mean obviously the cost of the service and what sort of care they are going to provide, personal care, and how regularly they can provide these services”.

(C-19)

#### 3.2.3. Proximity and Location

The proximity of older relatives to familiar surroundings, health professionals, family, or friends was highly relevant to participants. 

##### Familiar surroundings

Participants living far away were reassured to know that their parent was living in familiar surroundings such as their own home with carers visiting, or if they had to relocate that they were in a nursing home that was in a neighborhood that was familiar to them.

“I think location was top of the list…We wanted it to be close to where mum had lived so that she knew her surroundings and knew where she was, and it was not going to be so different…we did not want her to have another big shock”.

(C-14)

##### Near Familiar GPs and Health Professionals

It was also reassuring for many participants to know their parents were able to receive continuity of care from their usual GP and other health professionals.

“It [moving] would also mean that she would lose contact with her—the services that have been very important to her over many years such as her GP, her dentist, podiatrist, that type of thing. And I think also her friends”.

(C-17)

##### Near Family, Spouse and/or Friends

Staying in close proximity to other family members (not the participant), spouses, or friends was also considered important when the older parent lived close to them before being re-located to an aged care facility.

“[mother] had been really distressed about not being with dad, constantly asking where he was. So as soon as he said that he was willing to go into a nursing home, well that was just decided that she was going to go there too”.

(C-21)

“He has got friends within the village…he has got half a dozen friends whose wives have died, it is quite unusual; but they will …just drop in for a coffee, they bring him the paper and sit, talk. Q: So, am I right in thinking it is been a bit of a trade-off of not being close to family to enable him to stay closely attached to his friends? A: Yeah”.

(C-06)

An option participants considered was moving their parents closer to them. However, this was not viewed as an easy decision because adult children were aware that it may lead to confusion in their elderly relative due to them not being in their habitual environment or due to them being separated from other important people such as their partner.

“I would love Mum and Dad, and I tried to push for them to move up here. I sort of almost talked them into it. …my kids are here, my grandkids are here, I have retired here…But the problem is, is that Dad’s got all these doctors, and now with his dementia, he goes to the RSL, and he has his coffee with the boys that he used to be with the Lions with, and it is that sort of familiarity. Even when I bring him here at Christmas and stuff like that, he gets quite confused”.

(C-04)

#### 3.2.4. Reputation of the Care Provider

Participants who lived too far away to personally assess the quality of care provided by different services often relied on the opinions of trusted advisors such as family, friends, and professional aged care advisors who had experience with particular aged care providers. They were all viewed as a reliable source of information to guide participants’ decisions on aged care services.

“So, it became very apparent to me early in the piece that I needed help, so I phoned up and it’s the best thousand dollars I’ve ever spent in my life… I am just telling you straight up that they were a lifesaver for me. It was the [financial advice and placement service]”.

(C-20)

### 3.3. Factors That Influence Perceptions about the Quality of Care Provided

#### 3.3.1. Vigilant Monitoring and Responsivity

Participants spoke of their anxiety for the welfare of vulnerable parents because they could not monitor their parents’ wellbeing in person due to distance. This meant they placed great importance on aged care providers being vigilant with monitoring and responsive to the older person’s health and welfare.

##### Regular Assessment and Reporting

Participants generally wanted aged care facilities to be more proactive in their assessment and reporting of their older relatives’ health. There were some examples where the aged care providers had not noticed alarming deteriorations, and the older relative had not asked for additional help.

“I did actually say, when I was over there and I discovered that mum had been so ill, I actually spoke to [community carer] when she was there on the Friday and said, ‘Mum should have really rung up…and requested some extra help’. [community carer] said, ‘Yeah, she should have’. I said, ‘Next time, if you are seeing her like this, can you prompt her to do it?’ I think [community carer] would, I think they’ve got that sort of relationship now that she would”.

(C-15)

“I was really horrified her right leg was all hot and inflamed …I went and found the enrolled nurse and the RN came down together and had a look and said, ‘Okay’…We do not know if anyone is checking so that is why it helps to be nearby, and my brother is going in this afternoon. I said, “Can you check is anyone looking at her legs?” because if we lived—you know, if I lived a few hundred kilometers away like before, we would not even know her legs are swollen”.

(C-01)

##### Availability of Technology to Assist with Monitoring

Some participants were reassured by technology to help them monitor their parent’s wellbeing, such as Skype calls, where they could see the person as well as talk to them, and personal alert systems that enabled their parent to call for help in an emergency.

“I find skype is brilliant. There was a situation when dad was still at home… I think it was after another fall, he had a bandage on his wrist, and I would see him sitting in a chair in the background and I said, “What has happened to dad’s wrist?” Mum’s comment was, “You do not miss much, do you?” So yeah, I think skype is brilliant because it’s not only do you hear them, but you see them”.

(C-15)

##### Responsive to Family’s Concerns

The responsiveness of aged care providers to families’ concerns about care-related issues was highly valued. While some providers were deemed as being responsive, it took more time and effort from families to have issues addressed by other providers.

“Mum was complaining that someone had hold her, she had a bruised wrist, and she was saying it was one of the carers… so we just wanted that investigated a bit and so I did that communication with them by phone”. Q: “And how did that go? Were they responsive to your concern?” A: “Absolutely …they were onto it. So that was all done by email and phone, but I feel like I could text [residential care staff] and I would get a reply about anything. It is like I am there”.

(C-20)

“You have to hound them a fair bit to get stuff done. Stuff does not happen easily and that is what I mean in taking advantage, they do not—it feels like they hope you will go away”.

(C-03)

#### 3.3.2. Communication with Family

Participants were very reliant on and appreciative of the aged care providers keeping them up to date with their relatives’ situation.

##### Preference for Frequent, Regular Updates, Initiated by Service Provider and Contactable Staff

Participants expressed a strong desire for frequent, regular communication from the provider, in addition to notifications if problems have arisen. Ideally, they want services to take the initiative and communicate with them, rather than families having to ask for updates.

“If they did actually get back to me and say well, she is going well at this service. Or she is not. Or her Meals on Wheels did not come today, or just different things, that would be good…I thought she was going to this Wednesday social group…but then my mum was not going. So, no-one was picking her up. But I did not know that until I rang and said, can you just confirm how my mum is going? Is she settling in? It was up to me to pre-empt how she is going with everything. And then they came back and said, oh, she has not actually been coming”.

(C-19)

However, this was not a common occurrence. Only one participant spoke glowingly of his mother’s nursing home that communicated regularly with reassuring messages and photos.

“[Residential care facility] these guys get it right; I communicate a number of ways. I have the mobile phone number of the clinical nurse in charge of the site and also the client liaison person… and I have email addresses so a couple of things there like sometimes I get a nice surprise where [name] the RN in charge, would send me a photo of Mum with a budgie and that is wonderful”.

(C-20)

Participants also gave examples of difficulties contacting the relevant people within their parent’s aged care service. They wanted communication pathways that were easier to navigate, or a consistent contact person, especially with the added complexity of long-distance communication.

Information to be communicated includes changes to the wellbeing of their relative, services/activities available to their relative, and advice on navigation of the aged care system.

In addition to wanting to receive information about any changes to the health or wellbeing of their relatives, participants also reported that they often did not know what service choices and social activity options were available to their parents. They want aged care services to communicate these options to them so they could make informed decisions and encourage their parents’ participation in them.

“The staff…they do activities and they just put them on the board [in the nursing home] and sometimes that is really not enough for a lot of those people, they really need to be told about them and encouraged”.

(C-14)

Clear advice and assistance with navigating the aged care system would also be appreciated.

“Anything to do with something she does not understand, and quite frankly, the My Aged Care, and all the different people you get put onto, I am not a stupid person, but I get very annoyed and angry, because even the people I speak to, some of them do not know what is going on”.

(C-04)

#### 3.3.3. Flexibility and Proactiveness of Care

Participants appreciated those services that were flexible in the amount and types of care provided, and their proactivity in noticing and suggesting additional care options. 

##### Flexibility to Meet Changing Needs

The provider’s ability to provide additional services when needed and make these services flexible (e.g., in their type and duration) was highly valued by participants. When this flexibility was not offered, independence could be lost.

“Some of the things that when [the community care provider] works, it works well. But one that was reliable, consistent, was well-meaning, was caring, and was able to respond more quickly. Q: are you thinking that a service that could flex up and down in response to needs would be ideal? A: It would certainly be an improvement, absolutely. Yes”.

(C-17)

##### Proactive Attention

Participants would like providers to be proactive in offering services and care according to the parents’ needs. Unfortunately, not all services were proactive in noticing the need for different care options, leading to distress for the parent as well as their carer.

“They could see that mum was struggling with something or dad needed something they would suggest …’you have got funds there you have built up. You can have these little extra bits and pieces’—like the podiatry”.

(C-08)

#### 3.3.4. Staffing

All participants preferred caring staff that were consistent, skilled, available, trustworthy, and reliable. 

##### Continuity of Staff

Participants expressed their preference for carers who were familiar and had an ongoing relationship with their parents. Unfortunately, this was not possible in all cases with high staff rotation which caused disappointment in both participants and their parents.

“I like the way that she has the same person coming in all the time. So, there is [carers name] and mum have sort of built up quite a relationship.”.

(C-15)

##### High Level of Skills and Experience

Participants wanted to be able to trust the skills and experience of the aged care staff. They particularly valued the support provided by skilled nurses, with several examples given of parents requiring specialized nursing services (e.g., for wound care and colostomy care).

“[Father]…developed this cold …and then he got sicker and sicker… I just said to him, “I think I am going to ring the hospital…and he said, “I do not want to go into hospital, I hate going into hospital,” and I said, “Well you cannot be here on your own,” and luckily the carer, who is an ex-RN, probably the one that goes there the most, she arrived in the middle of it and she was very matter-of-fact about it”.

(C-06)

##### 24 h Nursing Care in Residential Facilities

There was an expectation of 24 h availability of skilled nurses in nursing homes.

“I was looking for too was a 24-h nursing care because one of the homes I went to they would call a nurse if they needed one”.

(C-01)

##### Honesty and Trustworthiness

Participants expected aged care providers to be honest and trustworthy with the care of their parents.

“A word just came into my mind which I think is important in relation to care and what I think is important. That word is trustworthy. I would like the person/persons who provide my mother with care to be caring and trustworthy”.

(C-17)

Similarly, participants expressed concerns about the safety of carers in supporting their parents’ daily activities. For example, the need for honest carers to help their parents with bank transactions or for carers to safely drive their parents to appointments, including while using their owning reliable vehicles.

“So, there would be different people turning up, there would be people who she considered to be inappropriate picking her up, people with inappropriately maintained vehicles that were driving her, people wanting to come into the house to use her facilities, and just generally very unreliable”.

(C-17)

##### Well-Coordinated, Reliable Care

Participants were reassured by evidence that the aged care service was well coordinated, resulting in reliable care.

“Mum was obviously a wanderer no matter where she was, so it became a challenge for them to manage. But one thing I liked the way they managed it…they have put a few engineering solutions in place. Stickers on doors… changing locks and they really put a lot of time and effort. Mum was about the only wanderer I think, of about 35–40 residents…But they put a lot of time and effort into sorting that out”.

(C-20)

##### Warmth, Caring, Respectful Attitude

Participants wanted aged care providers to show a warm, caring, respectful attitude to their parents. Unfortunately, there were several examples where participants had been troubled by the poor attitudes of staff, adding to their distress of being too far away to assist.

“I just felt like it was a very welcoming place and had a good rapport and relationship with [mother]… there’s a big family atmosphere in that home.”.

(C-20)

“I do not know, the staff they did not acknowledge me as I walked through… There was no sort of real connection with anyone as far as the staff was concerned. Dad was less than polite and called one of them a bitch… if the staff could not be bothered saying hello and helping me to find my father’s room, then what sort of care were they giving my father basically”.

(C-15)

#### 3.3.5. Access to Appropriate Holistic Care to Maintain Wellbeing

Participants spoke of wanting their parent to live in an environment that gave them easy access to a variety of services and factors to meet their wellbeing needs.

##### Appropriate Medical, Mental Health, and Allied Health Services

Participants wanted their parents to have easy access to reliable GPs, and allied health professionals such as podiatrists and physiotherapists. Examples were given of the parent’s inability to access rehabilitation following an acute episode such as stroke, which they found concerning.

“Well, she was in hospital for a while, then they sent her to an aged care place without any rehab …I think it was mainly the fact that she wasn’t getting any specific attention for the stuff with the stroke. She was not getting to walk; she wasn’t getting any physio…”.

(C-21)

“They have got a good GP there, that is important. The GP goes there regularly, and I just spoke to him the other day about Mum. I had a chance to talk to him about some issues with Mum, dementia, and he was really good… He knows Mum really well, knows her condition, I can tell that by talking to him”.

(C-20)

##### Appropriate Physical Environment

A number of environmental aspects of aged care facilities were relevant to participants. Appropriate access arrangements for people with physical disabilities, security, privacy, proximity to social and main service areas (e.g., office), and well maintained and aesthetically pleasing facilities were highlighted as relevant elements of care.

“And what sort of accommodation he would be in, whether it would his own private room or whether he’d be in a joint room and things like that. We have to consider all of those factors as well. He is quite a private person so I don’t think he would want to be in a share room style of thing”.

(C-08)

“It actually had the most beautiful north facing room where their patients could sit, and that beautiful winter sun would come in and it was quite a nice garden in front of it and it was really a lovely room…gardens were a main priority…her main interest and hobby was gardens and she had done botany at university, so she has had this love of trees and gardens”.

(C-14)

##### Appropriate Social Support

Social support was important to participants-friendly staff who would spend time talking to their parents, along with access to informal social activities and outings. Some participants wanted professional psycho-social support for their parents who they worried were depressed.

“Yeah, I think communication, the socialization, communication, having people who can chat and bring the outside world in is really important for morale, and if morale drops completely, that is the end of it”.

(C-06)

##### Other Aspects of Holistic Care

The relevant elements of holistic care differed across participants, illustrating the need for aged care providers to tailor services to meet individual needs. Examples included access to specific religious support, a garden, transport to appointments, personal care, nutritional support and housekeeping for those still in their own homes.

“…because we are a Christian family…I went to [residential aged care facility] and that’s just next to the [denomination] Church. Mum regularly attends the [denomination] Church so tick that box… They video stream – every Sunday they video stream the church service through into their meeting room onto the big screen”.

(C-01)

## 4. Discussion

This study highlights several factors remote carers consider when making decisions about aged care providers for their elder relatives. While many of these factors are also things that local carers would consider, their importance may be heightened due to the additional layer of complexity that challenges associated with travel (time, distance, and cost) and communicating from a distance, added for long-distance caregivers. For example, availability was found to be an important element when long-distance carers are selecting aged-care services, when the health of their elder relative is in rapid decline and there is pressure to transition from acute healthcare settings to aged care facilities or to return home with supports. While this is a challenge faced by families regardless of their location, in the case of long-distance caring, prompt availability may be extra important due to their inability to be present and perform caring tasks themselves, while alternative, longer term caring arrangements are being put in place. 

Like local carers [8], ensuring the costs of services are reasonable and affordable, and that elders are able to stay in their local area and/or near family, are other factors that are important to long-distance carers. However, the present research found new, additional location-related elements that were important to long-distance carers in the selection of aged care providers, such as the proximity to familiar GPs and health care professionals, and proximity to friends. Access to these trusted, long-terms supports is likely to be particularly important for this group, given their isolation from family members. 

This study also highlighted that long-distance carers often rely on information about the reputation of aged care services provided by aged care placement specialists, relatives, or friends, and use this to guide their decision making. This may be particularly important to them, given their inability to be present and make firsthand assessments of services. Previous research has identified the lack of appropriate information as a barrier in decision-making about aged care providers. Advertising seems to be the major source of information provided by services, but is often not trusted by families [8]. Together, these findings suggest there is a need to provide reliable information that supports aged care decision-making (particularly for those who cannot visit in person and do not have people they can call upon who know about the reputation of services). This may include the development and evaluation of long-distance care-specific information or decision making tools about aged care providers, which include results of assessments of aspects that are important to these carers, as identified in this study.

A number of factors that influence long-distance carers’ opinions of quality of care once a provider is selected were also identified by this study. Vigilant monitoring, timely and proactive assessment, as well as addressing and reporting on changes in the health status of elder relatives, were particularly relevant for long-distance carers. Understandably, it appears that their geographical distance from their relatives and associated inability to observe these changes in their relatives themselves, makes them feel particularly anxious and in need of careful reporting. Multiple examples of aged care providers failing to assess health changes, act upon them, and notify the family were identified in this study. Alternatively, long-distance caregivers valued regular, quality communications from service providers. 

Consistent with the findings in the present study on the importance of the honesty and the trustworthiness of staff, previous studies have found that due to the general lack of trust in the information provided to family members by aged care services, face to face visits are used to determine the actual quality of care provided [12]. To help address concerns, maintaining scheduled, regular contact with their elder relatives to keep updated on their health status, and the use of face-to-face visits to explore elders’ needs and the quality of the actual care being received is recommended [23]. However, it is acknowledge that this is not always possible due to geography, work, and family commitments.

Other characteristics of the staff (or formal carers) were also found to very important to the long-distance carers in this study. In addition to honesty and trustworthiness, they valued staff who continued in their role for long periods (to increase familiarity with the elders’ need, stability of care, and aid communication), who were skilled, experienced, and able to provide reliable care in a safe manner while showing a warm, caring, respectful attitude. Being friendly, warm, and skilled are also characteristics that older Australians and their (close) families value in aged care staff in general [8]. 

Having access to appropriate, holistic care in residential facilities was also considered important by long-distance carers in this study. Previous research has shown that having access to reliable medical care is also extremely important to Australian elders [5]. However, this study highlighted that this extends beyond having access to medical care for long distance carers—it is also important to them that their parents have access to broader wellbeing support, for example access to allied health and psychosocial services. This may be because they are unable to take them to these appointments themselves (unlike local carers). Aged care providers should seek to accommodate this and may benefit from highlighting this as a strength of their service when offering information to prospective clients. 

There are other important insights relating to communication that emerged from this study. Participants stated that ideally, communication (on issues such as changes to the wellbeing of the patient, services and activities that are available, and how to navigate the system) should be proactively initiated by the provider instead of the remote carer having to seek out information. Having a central, accessible contact would also be valued by long-distance caregivers. Agreeing on a communication plan at the start of service delivery may help set appropriate expectations and boundaries. Furthermore, greater use of technology by aged care providers to aid communication would be valued by many long distance caregivers. Previous research has shown that systems designed to manage information (e.g., client, funding and evaluation data) in aged care settings (especially in community aged care) tend to be primarily designed for and focused on the support of internal business processes and not on supporting client services and quality of care. This impacts on information provision to key external stakeholders and therefore care coordination and client safety [24]. A revision of the functionality of these systems may therefore be useful. New systems could perhaps be informed by communication tools currently being used in other contexts, such as schools to update external parties (namely parents) on the wellbeing of their children [25].

This study also highlighted that long-distance caregivers value aged care providers being flexible in their response to the needs of elder relatives and being proactive in offering new care services when deemed necessary. Aged care services should take into account the needs of the remote carers and the elder, while keeping in mind that care-related actions from professional or informal carers can at times limit older peoples’ freedom to make decisions (e.g., lack of appropriate mobility aids, and fixed mealtimes) [26,27]. 

This study focused on the experience of long-distance caring within the Australian health-care system. Previous studies have found different challenges for those caring for elderly relatives in transnational settings; for example, differences in culture and aged care systems that made it difficult to make decisions about aged care providers [12,28]. Future studies should consider exploring important aspects that influence decisions about aged care providers in transnational care settings, particularly in culturally and linguistically diverse populations, and learning from the experience of COVID-19. Most participants in this study were female. This limited generalizability; however, there is evidence that daughters more frequently fulfil the role of carers in the family [4]. Most of the study sample had a high education level (university and post-graduate degrees) and information about household income was not collected. Future quantitative studies could explore the influence of socioeconomic status in the selection of aged care providers by long distance carers for their elder relatives. It may also be useful to quantitatively compare the differences between the preferences held by those long distance caregivers with relatives in residential facilities versus home-based care, and measure differences (or similarities) in what is important to long-distance versus local carers.

## 5. Conclusions

In addition to the decisions faced by all carers responsible for elders accessing care, long distance carers appear to face additional challenges. There is the difficulty of accessing reliable information about the quality of care offered by aged care services, and the problem of receiving regular timely and accurate communication from the services about the wellbeing of the elders and the range of care options available. The availability, cost proximity/location, personal qualities of the staff, and access to holistic care were important. Future research should formally evaluate novel solutions to reduce isolation between all parties, particularly considering the impact of COVID-19 on family members’ ability to visit and the increasing number of people caring from a distance. The findings from this study could be used to develop tools to assist caregivers to discriminate between the different competing options when making choices about care, from a distance. They may also assist care providers to help ensure their services are orientated towards the needs of clients and their families who are caring from a distance and may therefore require additional support.

## Figures and Tables

**Table 1:** Characteristics of the sample and their older relatives.

Variable	Category	Number
**Characteristics of participants**		
Gender	Female	10
	Male	3
Age (mean years; range)		59.8 (50–65)
Relationship to the person receiving aged care services	Son	3
	Daughter	10
Employment status	Retired	3
	Working full time	6
	Working part-time	4
Highest education level	Secondary school	2
	University degree	6
	Post-graduate degree	5
Has been distant carer for two or more older relatives		5
Distance between participant and older relative (Google maps) (mean km; range)		1363 (384–3214)
**Characteristics of the older relatives (parents)**		
Living in own home with community services support		10
Living in residential care (high level)		6
Living in residential care (low level)		0
Age (mean years; range)		88.1 (78–98)
Time receiving aged care services (mean years; range)		3.9 (3 m–10 y)
Dementia or significant cognitive decline		6

**Table 2:** Thematic coding tree.

Themes	Interview ^1^	Sub-Themes
**Factors that influence decisions on an aged care service provider**		
Availability of care	6	
Financial arrangements	10	Cost
		Suitable payment options
		Value for money
Proximity and location	11	Familiar surroundings
		Near familiar general practitioners (GPs) and health professionals
		Near family, spouse, and/or friends
		
Reputation of care provider	7	
**Factors that influence perceptions about the quality of care provided**		
Vigilant monitoring and responsivity	11	Regular assessment and reporting
		Availability of technology to assist with monitoring
		Responsive to family’s concerns
Communication with family	13	Preference for frequent, regular updates, initiated by service provider and contactable staff
		Information to be communicated includes changes to the wellbeing of their relative, services/activities available to their relative, and advice on navigation of the aged care system
Flexibility and proactiveness of care	9	Flexibility to meet changing needs
		Proactive attention
Staffing	13	Continuity of staff
		High level of skill and experience
		Access to 24-h nursing care in residential facilities
		Honesty and trustworthiness
		Well-coordinated, reliable care
		Warmth, caring, respectful attitude
Access to appropriate, holistic care to maintain wellbeing	10	Appropriate medical, mental health and allied health services
		Appropriate physical environment
		Appropriate social supports
		Other aspects of holistic care

^1^ Number of interviews with data supporting the theme.

## Data Availability

Data supporting the findings in this study are included within the article.

## Supplementary Materials

The following is available online at https://www.mdpi.com/article/10.3390/ijerph182413000/s1, Table S1: Additional examples of data supporting the themes.

## Appendix A. Interview Guide

- Tell us about your experience caring for an older family member from afar
- Describe the care your family member is receiving
- What aspects of their care do you like?
- Because of living at a distance?
- What aspects do you think could be improved?
- What challenges have there been?
- Day to day; when the older person becomes ill?
- What choices have you faced about your relative’s care/the decisions you have had to make?
- The things you have had to weigh up when making these decisions?
- The impact of distance on the process?
- Describe your communication with the aged care provider
- What is most important to you about the care your relative receives?
- Is anything especially important because of distance?
