# Supplementary material for: Choosing and Managing Aged Care Services from Afar: What Matters to Australian Long-Distance Care Givers
Source: Int J Environ Res Public Health. 2021 Dec 9;18(24):13000. doi: 10.3390/ijerph182413000 (PMC8701222; doi:10.3390/ijerph182413000)
Supplement: Supplementary file 1 [file ijerph-18-13000-s001.zip › ijerph-1410605-supplementary.pdf]

Choosing and managing aged care services from afar: what matters to Australian long-distance care givers

Supplementary material

Table S1. Additional examples of data supporting the themes.

Themes	Sub-themes	Supporting data
**Availability of care**		At that time while she was still in hospital she was assessed… at high care permanent she qualified for on her assessment… But she's entered that nursing home as high care and the only room available was upstairs which was in the high care area, but cognitively she should really be downstairs (C-01)
		And then when [mother] passed away four years ago they said dad was entitled to help and could take on her share of the [community aged care package] (C-08)
		I think initially there was only one provider down there… There are now others, but they've only come in in recent years (C-06)
		Funding is an issue both in the community and residential care. Mum's bucket of money is OK at present, but as her care needs increase, that reserve will run out, and level 1 and 2 packages are not available when they are needed… There is a real gap in that level of care, which usually results in an admission to a nursing home from hospital, as it did for my father. (C-15)
**Financial arrangements**	Cost	Yeah, it needs to be, like I said, like some of the really nice places are, but you need a lot of money to be in them, and they don't have that. (C-04)
		You've got to consider the money factor as well. How long is his money, pension and everything going to last him in a nursing home, because they're not cheap. (C-08)
	Suitable payment options	And the billing – there were huge billing problems with [aged care provider], and she was getting very anxious about this, and I must confess it got to the stage where I was having to ring the person at [the provider] on Wednesday afternoons to check (C-17)
		…the money would be automatically debited from your account and that sort of stuff (C-01)

	Value for money	Q: So you looked at a few different providers, how did you go about choosing?<br>A: Geography and seemingly value for money in terms of admin fees versus delivery. (C-02)<br><br>Again, being of his generation, he used to go on about how much it was costing him… I think value for money, and I think despite what Dad was saying, when I saw the figures, I thought, they're so heavily subsidised, it's incredible value for money. (C-06)
**Proximity and location**	Familiar surroundings	I walked in the front door [of nursing home] and it's got these beautiful orange flowers which mum has in her garden at home. I went, oh, tick that box (C-01)<br><br>Well, location was reasonably close to where they lived so I think that was probably a reasonably important factor for them as well so the area was familiar (C-03)<br><br>I do know some of the staff that work there. And she [mother] does know some of the…residents. So some of – like her friends or her cousins. So that would be my first choice. (C-19)
	Near familiar General Practitioners and health professionals	So like I said, wanting them to come up here would have been great, but I think I've missed that window of opportunity, now they're much more reliant with their own doctors, and the Colostomy Association (C-04)<br><br>Yeah, and he's got a doctor …who is fantastic…she'll come into his home, she's spent a lot of time …she talks to him like he's got all his marbles…He's got such a good support set up there, the hospital's literally across the road from the unit. (C-06)
	Near family or spouse	It was just pure fluke once again that it was very close to us and my brother and his family lived only two streets away so they could walk to see mum (C-14)<br><br>We didn't know that he would end up in the same facility as Mum but it was going to be a matter of where there was a bed within southern Tasmania.<br>Q: Was that a strong preference anyway, to keep them close together?<br>A: Yes, I think so. I believe so. (C-23)
	Near friends	But she's got one friend that's still alive in [town] and she's on her own as well…They go out to do their shopping on a Sunday together and they go to a café and have a coffee first, as a bit of a sort of a routine around that and that's sort of holding her in [town] I think. But if [the friend] were to die, then I think mum would happily move very quickly. (C-15)

**Reputation of care provider**	We were happy to go with [residential care provider] I think because my husband's mother had been in there for many, many years so we knew a little bit about it and we knew it was quite a nice environment and the people were friendly and polite… the other thing that helped us was we did know someone that worked there and we did speak to her and she is from the area and she knew the other place too… and she was quite helpful in helping us, she knew it well so she could tell us a few things. (C-14)<br><br>…my mother's Uniting Church minister said they've [residential care provider] got a good reputation. My daughter who works as an OT said they've got a good reputation… I think also with all the bad press about certain nursing homes we had on the media and TV and all that I haven't heard a bad word about [residential care provider] anywhere. (C-01)<br><br>[residential care provider] seemed to be the most prominent care group down there in [regional area]… It just seemed to be the better one that was down there, the most prominent one out of the lot. And we've had absolutely no complaints about them at all. They've been absolutely fantastic. (C-08)<br>Seniors Information Service…were the best source of information, I just do not know how we would have got through those initial periods without them…They found us two really good people, a broker service and…a wonderful financial advisor who we just loved, totally in sorting money problems in this sort of situation and so we had a couple of roundtable discussions… (C-14)<br><br>You've got to appreciate other people's input in what they see and – because outsiders we don't know what's available out there for oldies. So it's a learning curve for all of us so they have just, the women coming in and seeing what needs to be done and what can be done is a God send, really. (C-08)<br><br>So it became very apparent to me early in the piece that I needed help so I phoned up and it's the best thousand dollars I've ever spent in my life... I'm just telling you straight up that they were a lifesaver for me. It was the [financial advice and placement service]. (C-20)
**Vigilant monitoring and responsivity**	He had somebody coming in morning and night to make sure he was okay and coping. And there were people who then, if there were problems, they know what to do…But it is that distance that makes that very important. (C-06)<br>They certainly just kept an eye on what was going on and if they saw anything that might have helped in any way they were forthcoming, very much so, in helping them out with extra bits.

	Q1: And because you're at a distance is that why you think that you valued that sort of oversight in particular or -<br>A: Definitely, yes (C-08)
Regular assessment and reporting	We drove through quickly [to visit mother] …because we thought we need to get there, something is wrong…And on the way there I had already emailed [aged care provider] …I just emailed them and said, "Why hasn't somebody noticed that she's ill?" pretty much. You know, just quite politely. We do rely on them being there twice a week to sort of, you know, keep an eye out. (C-01)<br>… I was really horrified her right leg was all hot and inflamed …I went and found the enrolled nurse and the RN came down together and had a look and said, "Okay."…We don't know if anyone is checking so that's why it helps to be nearby and my brother is going in this afternoon. I said, "Can you check is anyone looking at her legs?" because if we lived – you know, if I lived a few hundred kays away like before we wouldn't even know her legs are swollen. (C-01)<br><br>We are very relieved to know that he has somebody coming in morning and night, but the days that he's on his own during the day, when nobody's visiting …he's got mates in the village, so we know that there's somebody looking in on him at least every 10 to 12 hours…Which from a distance, and from my point of view especially, is incredibly reassuring. (C-06)
Availability of technology to assist with monitoring	She's got a safety – what's it, a call alert thing …which she wears at all times, which is great because she's not one of these people that leaves it one the bedside table when she showers or something… with the safety alert, she's got to ring in every month to make sure that that's still operable, to make sure that's okay. (C-15)
Responsive to family's concerns	There was an occasion where we had an issue with one of the cleaners… instead of cleaning the house she decided that she was going to wash my mother's hair and curl it. ..And so, all right, I get on the phone to [community care provider] and they said, "Are you sure?" and I'm, "Yeah." And anyway they said, "We'll deal with this and we'll talk to our carer" So they sorted that one out for us and then I think my sister stayed a few weeks later and the house was all clean again. (C-01)<br><br>I was quite happy with the care dad got. I mean, he had a couple of grumbles but when we brought that to the site manager's attention, they were fixed very quickly. So no, I mean if they knew that a resident had a problem they tried to resolve it as quickly as they could. So from that point of view no, I mean it was good, yeah. (C-15)

		And then on a Thursday… the carer actually comes into her home and cooks a meal in her home, with – she pre-buys the things. And then she comes into her home and cooks for her. So keeps her company while she's cooking, because she gets Meals on Wheels the other days of the week.<br>Q: Was that something that was important to your mum to have some cooking in the house?<br>A: It was important to me. So I actually brought it up with the carers. (C-19)
	NOT responsive example	Q: Just at the moment, you mentioned that your mum's unwell. Is this service able to respond to that? Like, a sudden health crisis?<br>A: Well, no. She doesn't feel well enough to go to the doctor's… she doesn't want to go outside. She's hoping her GP will be able to do a home visit tomorrow, and so it's thinking about transport if she had to go to the surgery. There's no-one that can do that at the moment...the pharmacist is coming in tonight and so she's worried about asking him to go in two days in a row if she needs a prescription filled. I don't know that [community care] would respond to those types of things. (C-17)<br>But at the beginning, whenever I went there, which I tried to get there every four to six weeks, there was new people there so you never knew who you were talking to and who had told this person that. I did things like type up some instructions or some suggestions to help integrate mum, you know, I laminated them and put them up but it was just really ad hoc. (C-21)
**Communication with family**		Q: how do you feel about the communication systems that seem to be in place and whether that would work for you and your sisters to keep up to date with what's going on?<br>A: It remains to be seen. I'm quite open to ringing and talking to them about things, if Dad says something that sounds a bit strange or whatever. [My sister] made the comment she felt a bit like the squeaky wheel…she was just asking questions, if you never ask, you'll just wonder. She felt a little bit that they'd see her coming and think oh, God, here she comes again. (C-21)<br><br>There are quite a few staff there who have actually got country backgrounds and they really understand the difficulties we have and just what it's all about to live in the country ….they do always text or ring if mum has got the urinary tract infection, they will just ring and let me know that she's going onto antibiotics but I would have thought that was fairly standard. (C-14)<br><br>I think what was a bit frustrating is when things don't work, I'm up here, and they're down there. And Mum might tell me something, but it's then trying to get through to somebody, and you get put on hold, then you've got to ring this number if it's about this, or this is a different person. (C-04)

Family alerted to changes	As baby boomers start to need community and aged care services.. we are also a generation whose children live far and wide. I have two kids in London and our third is in Sydney…The health care and community care system needs to be robust enough to spot [deterioration or changing needs], inform our NOK's of the situation and advocate for our wellbeing. (C-15)<br><br>Q: Is there anything else they could do to make it easier for you to feel involved in the planning of your mum's care from a distance?<br>A: I think …even email communication as to what's happening there and I don't mean a monthly newsletter as to what's happening in the whole centre, I mean specifically what is going on with my mum. (C-03)
Frequent, regular communication/ updates	Well you know, a lot better communication I'd say and, you know, I guess that's up to me as well but I only ever get a call if he's had a fall or if he's ended up in hospital.<br>Q: What sorts of things would you like to be informed about?<br>A: Just how he's going on a daily basis. Not daily but a regular update I suppose because, you know, I can't speak to him on the phone anymore, he just can't hear for a start and I can't ring him because he hasn't got a phone in his room. (C-21)<br><br>Q: How does the facility communicate with you …regarding anything to do with your parents?<br>A: We haven't had anything like that as yet apart from financial stuff…<br>There was another friend down there whose mother is in the same institution…she commented that they do have an annual - they offer you an annual meeting (C-23)
Information on available services and activities	So how often are they checking once they know there's an issue? Is there a set up? There may be, but we don't know. So we're still finding out things like that. (C-01)<br><br>With me at a distance?... apart from getting us together physically and sitting down in a room and discussing options, that of course would have worked really well, but I guess maybe if there was a person who had been able to set up a teleconference or liaise between the three of us, that would have been useful. (C-17)<br><br>OK so they said 'you've got some money left over so we can put that to that'. Well if she's got money left over, why haven't they already flagged that with her, so she can be using it? (C-03)

**Flexibility and proactiveness of care**	Flexibility to meet changing needs	I know when mum wanted to get a gardener in to do regular gardening, she enquired, I think, through them and they said to her 'yes, you have got some money left over at the moment in your [care package]' (C-03)<br><br>Yes, she has services seven days a week. Varying amounts of time. It could be something three days a week, it's only half an hour. Just for a shower and a general tidy up in the kitchen, make sure she's had her breakfast. That sort of thing. The other days it's shopping, cooking, cleaning. So other days could be two and a half hours a week. (C-19)
	Lack of flexibility examples	Well with dad, because he was still independent he found the restrictions really difficult to take, you know, being independent and then going into a place where he had to obey the rules and they told him when he had to get up and when he had to wash and whether he could have a drink or not have a drink. Yeah, so that stuff was hard for him. (C-21)<br><br>…the funding will now follow the person rather than stay with a provider. But in terms of having more flexibility like, for example, Mum got ACATed as Level 4 but if there still isn't someone that can give you Level 4 you still can't have a Level 4. The government haven't funded as many positions as what the ACAT people pump out. (C-02)
	Proactive attention	They were forthcoming when it came to what mum and dad were allowed to have with the allowance that they got…if the women came into the house and they could see that mum was struggling with something or dad needed something they would to suggest …"you've got funds there you've built up." You can have these little extra bits and pieces – like the podiatry. (C-08)<br><br>I'd like them to be more proactive and just suggest these things that I've suggested to them. Like coming to the home to feed my mum – just following up on things so I don't have to. That would be really helpful. (C-19)
	Examples of not proactive attention	If I'd actually been there and seen her I would have been a lot more alert as to how sick she really was… she [mother] showed me one of her statements and she had $1,700 that she had available to spend, if you like. And I said, Now that money could have been used, you know, it's there for you and this is the exactly the time that you can draw on that money and say now can someone come round, do some shopping for me, cook me some soup, cook me some meals that I can have in the fridge or the freezer and that would have helped you along no end. (C-15)

		That was one of the things, in the aged care home she'd be banging on the bed because she wouldn't understand to press the buzzer and she'd be there for ages before anyone came to find out what it was that she needed. (C-21)
**Staffing**		Q: [is there]…about your father's aged care that you think is difficult or important because you're at such a distance?<br>A: Well communication is important, and to me it's just that safety net emotionally for me knowing that he's got good quality care, regularly, frequently, and he's physically and pretty much emotionally – as I say, he's been very down since he went into hospital, but at 95 I don't blame him, you know? Life's an effort, everything's an effort, so that side of it, nobody's going to change that. I think from a distance it's just that knowledge that he's safe. (C-06)
	Continuity of staff	I think dedicated case-carers who have a bit of history with the person and knowing what's going on in their life. Maybe more contact with them, I'm not sure how often the contact is now. (C-03)<br><br>Just having a one contact person maybe, as a – I mean, Mum originally had a case worker, which was great, but then they left<br>Q: Was that easier, when there was a case worker that you could contact?<br>A: Absolutely. Because they actually knew Mum. They knew what was happening, or they would have a file in front of them. When you talk to people now, I mean they've got no idea (C-04)<br><br>But if they're short-staffed, they sometimes ask in agency nurses down from town…he doesn't like it when somebody comes in who really has no idea what to do and he's got to…explain everything, tell them what to do. But his regular carers know him well, they look after him, they're company, they cheer him up, they chat. (C-06)<br><br>The nursing home does seem to have a whole lot of different staff. There must be a lot of casuals or whatever, so that it's not always the same people, so that upsets my dad a bit, I think. He gets used to a couple of people and then it's someone else. (C-23)
	High level of skill and experience	He's had several medical problems with pressure sores and stuff, so the nursing side of it has kicked in occasionally, and that has been very good…The carers are good, they'll flag anything to the office if

	there's anything they see that's not that should be, but I'm not sure that all of them would know when to flag. (C-06)
	I'd want to make sure that there was – the staff were all well-trained. I'm actually – well, you know I'm a pharmacist (C-17)
	…she also then had a colostomy that she didn't have originally… lots of complications. We also had pulmonary oedema…all sorts of things happened that went wrong. So we then needed a nurse to come in and check her wound, we needed somebody to come in and help with the colostomy to start off with, and somebody to help shower, home cleaning. (C-04)
Access to 24 hour nursing care in residential facilities	I was looking for too was a 24-hour nursing care because one of the homes I went to they would call a nurse if they needed one. (C-01)
	I would like to think there'd be 24-hour registered nurse cover, and I know that's not always the case. (C-17)
	There's not enough RNs there…if Dad gets stroppy they go and report to the RN and then they advise them what to do, and they generally just back off. They don't force him to do things.<br>Q: Do you think that's important for your father to have always access to an RN?<br>A: Well, I think so, but Dad's not that hugely unwell yet. That might become an issue later. I just know in general, if I was looking - if you go looking for an RN, they're hard to find. (C-23)
Honesty and trustworthiness	We don't live nearby we do randomly visit and we are not telling the carers that we're visiting because, you know, she's our mum we'll just rock up and see if they're doing their job. We rock up and visit mum but we also look around. You know, just being honest that's what we do. (C-01)
	Aged care is given a bad wrap due to a few dodgy providers. The majority of homes and the staff do their best to support residents to maintain their independence as much as possible whilst providing the support that the person needs.(C-15)
	I must confess it got to the stage where I was having to ring the person at [community care provider] on Wednesday afternoons to check to see whether someone was actually going to pick her up on Thursday morning to do her shopping. And I'd be assured that someone would be there, and then they wouldn't turn up or they were late. (C-17)

		…she doesn't know how to use a key card or a card,…or how to use an ATM, but she doesn't get it. So she still goes into the bank and she writes out a cheque to cash…Which means someone has to take her to the bank to get her cheque to get the money and that person would generally be a carer. So you have to trust that they are giving her all the money back that she's getting out. (C-19)<br><br>Someone would come in a couple of times a week. It was mainly because he had a lot of falls so I was mainly worried about someone being able to keep an eye on him and be there. …I was anxious about him because of the falls. We did put in place the Red Cross thing where they phoned him every morning. But a couple of times he fell and couldn't get up and was there for quite some time (C-21)
	Well-coordinated, reliable care	But they seemed to be very well organised with their carers and their staff. They told me – there was a guy there he was in charge of hospitality and meals and all of that and, "If you've got any questions about that see him. If you've got any questions about admin see the lady [name] in the office. (C-01)<br><br>Q: what would be ideal for your mother and you?<br>A: Well, my mother needs reliable support. (C-17)
	Warmth, caring, respectful attitude	Yeah, it was obvious he really liked her [the carer] , you know, that felt like it wasn't an impersonal service, it was someone who really cared about what was going on.<br>Q: So why do you think he really liked her, do you know?<br>A: Because she obviously cared about him, she'd talk to him. (C-21)<br><br>I think the most important thing is that the staff are respectful and will give a little bit of time to the patients and have a joke or quick chat with them, I think it's always underestimated how much a smile or a quick hello, just a pop-in, head popping in the door and saying 'hello, how are you today?' is so, so valuable. It is such a boring existence and if the staff are just racing past your door all the time just doing their duties that doesn't, to me, constitute care. (C-14)<br><br>And certainly the women that I've met over my time being backwards and forwards have all been wonderful people. Very, very caring towards mum and dad. Really, really nice people. So we have no complaints. (C-08)
**Access to appropriate, holistic care to maintain wellbeing**		If you think about it, we keep talking about holistic care, and giving the choices back to the client, the family, and all the rest of it, but it is still very disjointed. We're not linked in, we're not sharing information so that it is that holistic, "Let's look at the whole person," they're all not involved with it, because we're not all part of a team. (C-04)

Appropriate medical, mental health and allied health services	The other thing is, I feel they don't get enough mental health support. Mum was very depressed when she came home …and my Dad is quite depressed at the moment, and that's causing issues for Mum. (C-04)<br><br>Well the valuable things are the things that are essential so that's her medication patch (C-02)
Appropriate physical environment	
- Access	And the ease of access to get because she'd never had any walking aids before and then she left hospital with a walking frame so I was looking at is there a ramp? (C-01)
- Psychosocial needs	She also had a fear of being a long way isolated in an area away from the office so it's actually been her main request…that she be near the office where there is a hub of activity (C-14)
- Security	She's a wanderer. A prolific wanderer. They had doors there which stayed open a long time…electronic doors actually stayed open for a long time before they shut. So that night, that first night I got home, I thought 'brilliant we've placed her' and then I got a call about 10 o'clock that Mum had gone missing. (C-20)
- Privacy	He was in a shared room with someone who had dementia and the other guy he was with was quite disruptive and kept trying to climb into dad's bed and there were other people, sort of kept coming into his room and it was quite distressing for him being in that situation. (C-15)
- Well maintained	I mean, the bricks and mortar have to be sound and clean. (C-17)
- Aesthetics	
Appropriate social supports	I tried to organise someone who was just a companion, someone who would just go there every day or a few times a week to just chat with him, just talking. I mean I think the loneliness is the thing because in the end he couldn't get out of the house. That was the hardest thing. I couldn't manage to organise anything like that. (C-21)<br><br>…he was sort of at a dining room area with people that could actually converse with him and he could actually talk to and what have you. He'd ended up going on the resident committee…So yeah, you know, he was – he hated being in the nursing home he wanted to be home with mum… I mean,

	it didn’t resolve the fact that he wanted to go home, but it certainly gave him something else to think about. (C-15)
Other aspects of holistic care	
- Transport	I know she does enjoy the excursions and she wasn’t able to do them until this year I think because she is in a wheelchair, she has got one leg now and the bus that they had couldn’t take her but they have got a new bus this year so now she can go on it but I think she does actually like that. (C=03)<br><br>But once that transport becomes an issue, they are quite isolated…but mobility with Dad, he can't walk too far at all, he's really bad (C-04)
- Nutrition	The food is really good. I’ve sat down at dinnertime and lunchtime with Mum and the food looks great…. Mum has never looked healthier, I’ll say that. For the last 18 months of Dad’s life, poor old Dad he was the cook, but he didn’t cook anymore so they were eating takeaway (C-20)<br><br>No, she doesn’t cook anymore. She’s getting meals delivered.(C-03)
- Personal care and housekeeping	It could be something three days a week, it’s only half an hour. Just for a shower and a general tidy up in the kitchen, make sure she’s had her breakfast. (C-19)<br><br>…after she [mother] died we persuaded him [father] to start getting somebody in two or three nights a week to help him get changed for bed, because that's a problem…Obviously the physical care is important, their ability, the quality of that physical care. (C-06)
